# Leachability and Health Risk Assessment of Cadmium and Other Heavy Metals in Agricultural Soils from the Mae Tao Watershed, Northern Thailand

**DOI:** 10.3390/toxics13080687

**Published:** 2025-08-18

**Authors:** Nipada Santha, Thanan Watcharamai, Rungroj Benjakul, Schradh Saenton

**Affiliations:** 1 Department of Geological Sciences, Faculty of Science, Chiang Mai University, Chiang Mai 50200, Thailand; nipada.santha@cmu.ac.th (N.S.); t.watcharamai@gmail.com (T.W.); rungroj.b@cmu.ac.th (R.B.); 2 Environmental Science Research Center, Faculty of Science, Chiang Mai University, Chiang Mai 50200, Thailand; 3 Advanced Research Center for Computational Simulation, Faculty of Science, Chiang Mai University, Chiang Mai 50200, Thailand

**Keywords:** heavy metals, agricultural soils, health risk assessment, Mae Tao watershed, leachability

## Abstract

Decades of unregulated zinc mining activities in the Mae Tao watershed, located in Mae Sot District, Tak Province, northern Thailand, have resulted in the pervasive contamination of agricultural soils with heavy metals, particularly cadmium (Cd), zinc (Zn), lead (Pb), and manganese (Mn). This legacy pollution has significantly impacted multiple environmental compartments—including surface water, groundwater, and sediments—and poses chronic health risks to local populations. This study investigates the key geochemical and physicochemical factors governing the leachability and mobility of these metals from contaminated soils and evaluates the associated human health risks. Controlled leaching experiments demonstrated that ionic strength exerts a more pronounced influence on metal mobilization than pH or other tested variables, suggesting that the electrolyte composition of pore water plays a dominant role in heavy metal transport. Despite elevated total concentrations of Cd, Zn, Pb, and Mn in the soils, hazard quotient (HQ) calculations indicated no significant non-carcinogenic risk under typical exposure scenarios. However, Cd exhibited a carcinogenic risk above the acceptable threshold at both average and peak soil concentrations, underscoring its potential to adversely affect human health. These findings enhance the understanding of heavy metal behavior in contaminated agroecosystems and provide a scientific basis for targeted risk management and long-term monitoring strategies in the Mae Sot region.

## 1. Introduction

Heavy metal contamination in soils has been one of the major environmental problems leading to several detrimental concerns regarding the environment and human health [1,2,3]. Heavy metals are released into the environment from the dissolution of primary sources such as minerals and rocks, or they may be leached from soils and sediments from sorbed metal ions, which are called a secondary source. The released metal ions are toxic and may pose a human health risk after prolonged exposure. Health risks arising from heavy metal exposure can be intensified in areas with industrial operations [4], dumpsites [4], and mining activities, including mining operations, smelting activity, mine waste, and tailings disposal [5,6,7]. Heavy metal pollution in agricultural soils has been widely reported across various regions. In African contexts, particularly within the six geopolitical zones of Nigeria, the literature indicates that the primary sources of heavy metals such as Cd, Cu, Ni, Pb, Zn, Co, Cr, Fe, and As include urban and industrial effluents, deteriorating sewage infrastructure, sewage sludge, fertilizers, and pesticides [4]. The concentrations of these metals span a wide range, with maximum levels frequently exceeding the WHO’s permissible limits [4]. Their presence in agricultural soils poses serious environmental and public health concerns, as evidenced by their accumulation in crops, cow’s milk, and animal organs [8,9,10,11]. Similarly to Kafr El-Zayat—a major agricultural and industrial city in Egypt—which faces significant soil contamination, particularly with aluminum, arsenic, cadmium, chromium, and nickel [12], these metals are known carcinogens and can cause damage to the brain, kidneys, liver, and other organs, primarily by inducing oxidative stress through free radical generation [12,13,14]. Furthermore, a review of heavy metal contamination in agricultural soils resulting from mining activities identified 72 affected mining areas in the southern and eastern regions of China [15]. The results indicated potential non-carcinogenic risks from the ingestion and dermal absorption of As, Cd, Cr, Cu, Ni, Pb, Zn, and Hg, as well as potential carcinogenic risks from As [15]. It was observed that non-carcinogenic risks were especially associated with lead–zinc, manganese, and tungsten mines, more so than with copper, gold, and iron mines [15]. These findings highlight that heavy metals in agricultural soils contribute to increasing toxicity levels and elevated public health risks, as evidenced by bioaccumulation and biomagnification.

The Pa Daeng zinc mine, located in Mae Tao watershed, Mae Sot district, Tak province, northern Thailand, is the largest zinc mine in Southeast Asia, which has operated for almost four decades. The mining activity continuously feeds cadmium, zinc, and lead contamination into nearby surface water, groundwater, and soils [16,17]. The Pa Daeng zinc deposit is characterized as a secondary mineral deposit [18], which was formed via the infiltration of zinc solution as a result of oxidized primary zinc sulfide and subsequent groundwater transport before it was redeposited along the fault planes, fractures, and pore spaces in the rock [19]. The redeposited ore was weathered by rain, surface water, and groundwater, thus generating a continuing flux of dissolved heavy metals. The Mae Tao Creek and its tributaries are the major transport route of the sediments and have contributed to the distribution of heavy metals in soils and sediments downstream [20]. Moreover, the Mae Tao Creek system is the main recharge into the Mae Tao watershed, making it a chief water supply for local people for drinking, consuming, and agriculture [17]. The Mae Tao watershed, which is close to a mining area and human communities, could cause environmental and health issues.

Over almost two decades, there have been numerous studies on health-related and environmental problems in the Mae Tao watershed. It has been revealed that this area has a wide range contaminants, such as cadmium, zinc, lead, and manganese in soil, water, and plants. The most dangerous situation is that the high mobility of cadmium and zinc in soil–plant systems permits their easy entry into food chains [21], where they may stimulate both human diseases [22] and toxic effects on animals, microorganisms, and plants [23]. The harmful health impacts on cadmium-exposed populations in the Mae Sot district have been reported [24,25,26,27,28]. Contamination has led to health risk assessment, where it has been reported that Mae Sot residents have irregularly higher cadmium contamination in their blood, bones, and urine than normal Thai residents, resulting in a 10–86% increase in disability-adjusted life years [29]. Recently, it was found that Mae Tao’s rice can take up heavy metals, which accumulate in the grain [30,31,32], and that rice consumption causes health impacts. Almost 20% of 159 samples collected in six villages of the Mae Tao sub-district, Mae Sot district, Tak province, were contaminated with a high content of Cd [30]. Sticky rice contained, on average, 0.1–0.7 mg/kg of Cd, with 2.60 mg/kg as the maximum value, while white rice showed 0.1–0.3 mg/kg, with the maximum at 1.94 mg/kg [30]. A health risk assessment of rice consumption was conducted and the result identified was that both types were regarded as having a significant hazard quotient (>1) at the maximum Cd concentration; however, only sticky rice poses a health risk when considering the average Cd content [30]. The stream sediments of the Mae Tao Creek showed hazardous levels of cadmium and zinc, with maxima of 37.11 mg/kg and 1231 mg/kg, and the highest values of suspended solids were found to be 18.27 mg/kg of Cd and 17,767 mg/kg of Zn [17]. Intense concentrations of Cd and Zn in the soil of the agricultural area receiving water from the Mae Tao Creek were found—5.93 to 30.4 mg/kg and 286 to 594 mg/kg, respectively [32]. Heavy metal contamination in the Mae Tao watershed has led to a particular focus on health risk assessments of rice consumption, but the persistence of solid concentrations in soil and stream sediments that function as the main heavy metal source for plant uptake, to which humans are directly exposed, have been considered only in concentration and distribution. Therefore, potential health risk assessments of heavy metals in the agricultural soil of the Mae Tao watershed require further study.

Heavy metal contamination can be determined from its intensity and its leaching potential, as well as chemical speciation, since the toxicity of heavy metals depends on the concentrations of species [33,34]. Heavy metals will be more harmful when they are leaching and being distributed into biota (bioavailability). John and Leventhal [35] gave the definition of bioavailability as the ratio of total element concentrations that are available for integration into biota or bioaccumulation [35]. They also explained that the plant root can take up the heavy metals crossing the membrane of epidermal cells into plants’ cells (bioaccumulation) from the contaminated soil; afterwards, the metal accumulated in plants is consumed by herbivores and humans [35]. In this case, the trace elements enter through the food chain and accumulate in the higher-order consumer, which causes significant health risks across multiple physiological systems [36]. For instance, cadmium exposure is extensively linked to a range of adverse outcomes, including respiratory issues like lung diseases, hypertension, and various malignancies such as prostate, bladder, pancreatic, kidney, and breast cancers [37]. Furthermore, Cd has been implicated in neurological disorders, notably Alzheimer’s and Parkinson’s diseases [38,39,40]. Its ability to cross the placental barrier also raises concerns about its potential impact on fetal development [41]. Similarly, lead exposure is known to disrupt a wide array of bodily functions, affecting the neurological, skeletal, reproductive, hematopoietic, renal, and cardiovascular systems [42]. While essential in moderate amounts, excessive zinc intake can lead to toxicity, manifesting as adverse effects on the neuronal, gastrointestinal, or respiratory systems [43]. Likewise, chronic overexposure to manganese can result in progressive and permanent neurotoxicity, often accompanied by toxicity to the lungs, heart, liver, and reproductive system [44,45]. Therefore, the bioavailability of heavy metals that have been identified as having a significant impact on human health will be used for potential health risk assessments.

The primary objectives of this study are to characterize the geochemical properties of native soils and quantify the total concentrations of cadmium (Cd), zinc (Zn), lead (Pb), and manganese (Mn). Soil samples exhibiting elevated levels of these metals will be further evaluated for their leachability under controlled conditions, with a particular focus on pH and ionic strength—two key factors influencing metal bioavailability [35]. This assessment aims to elucidate the potential for metal mobilization within the environment. In addition, a comprehensive human health risk assessment will be conducted by considering all relevant exposure pathways associated with the use of contaminated agricultural soils.

## 2. Materials and Methods

### 2.1. Study Area

**Figure 1:**
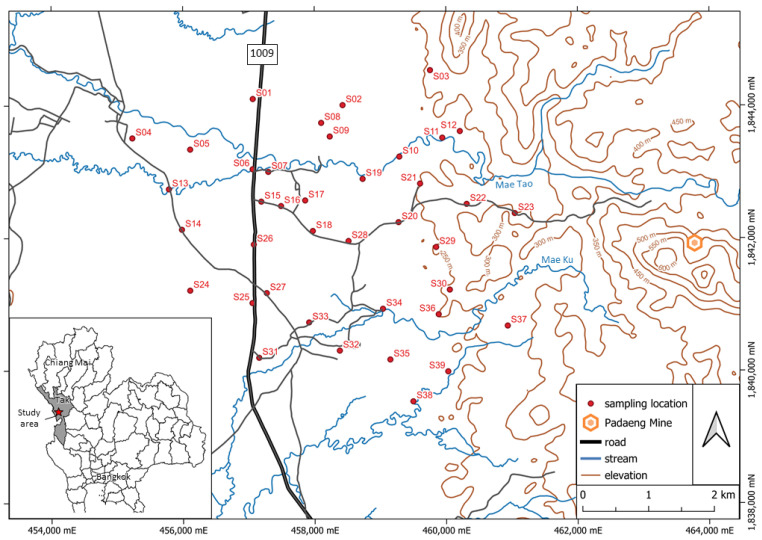
The locations of sampling points in the study area and Pa Daeng zinc mine, Mae Tao watershed (modified from Royal Thai Survey Department, 1999 [46]).

Soil sampling was conducted within the Mae Tao watershed, an agricultural region located in Mae Sot District, Tak Province, northern Thailand. From an environmental geology perspective, this area has been identified as a hotspot for heavy metal contamination, particularly along the Mae Tao Creek, which flows downstream from the Pa Daeng zinc mine [17] (see Figure 1). The creek also serves as a primary source of irrigation water for local agriculture. This study aimed not only to assess the total metal concentrations, but also to evaluate the bioavailability of heavy metals in agricultural soils. The sampling design was primarily based on land use classification, with soil collected specifically from paddy fields currently used by local farmers for cultivating rice and corn.

### 2.2. Field Sampling

A total of 39 agricultural soil samples were collected across an area of approximately 25 km^2^. Sampling locations were selected randomly from active farmland currently used by local residents for cultivating corn and rice. To minimize spatial autocorrelation, each sampling point was spaced at least 200 m apart, as illustrated in Figure 1. The sampling sites were georeferenced based on the topographic map of Mae Sot District (Sheet 4742 III) [46]. Soil samples were collected from a depth of 30 cm below the ground surface, which covered the plowing depth in the study area, using a spade. Approximately 1 kg of soil was placed into clean, transparent polyethylene zipper bags while maintaining field-moist conditions. The bags were sealed immediately after sampling to preserve the natural moisture and chemical properties of the soil, as required for subsequent geochemical and physicochemical analyses in the laboratory.

### 2.3. Chemical Analysis

#### 2.3.1. Basic Soil Properties Analysis

The basic soil properties were determined using a 1:2 soil-to-water suspension method [47]. For each sample, 50 g of soil was placed into a 250 mL flask, followed by the addition of 100 mL of distilled water. The flasks were sealed with Parafilm^®^ (Bemis Company, Inc., Neenah, WI, USA) to prevent evaporation and contamination. Measurements of key soil parameters—temperature (°C), pH (hydrogen ion activity), electrical conductivity (EC, in µS/cm), and oxidation-reduction potential (ORP, in mV)—were conducted at multiple time intervals: 0.5, 1.5, 4, 12, 24, 36, and 48 h after mixing. The pH and oxidation-reduction potential (ORP) were measured using a portable pH meter (HI9125, Hanna Instruments, Cluj Napoca, Romania), while electrical conductivity (EC) was determined with a multi-range conductivity meter (HI8733, Hanna Instruments, Amorim, Portugal). These measurements provided insights into the temporal stability and interaction of soil’s chemical properties in an aqueous suspension.

#### 2.3.2. Chemical Analysis for Total Element Concentration

Prior to chemical analysis for the total elemental concentrations of four heavy metals—cadmium (Cd), zinc (Zn), lead (Pb), and manganese (Mn)—approximately 500 g of each soil sample was oven-dried at 70 °C for 72 h. Once dried, samples were manually cleaned to remove extraneous materials such as rock fragments, plant residues, and other debris. The cleaned soil was then finely ground using a porcelain mortar and pestle to obtain a homogenous powder. Each powdered sample was divided into two subsamples: one sieved through an 80-mesh nylon sieve (<177 μm) and the other through a 200-mesh nylon sieve (<74 μm).

Subsequently, approximately 500 mg of each sieved subsample was subjected to acid digestion using a tri-acid mixture comprising 20 mL of concentrated nitric acid (HNO_3_), 20 mL of hydrofluoric acid (HF), and 2 mL of perchloric acid (HClO_4_) in a ratio of 10:10:1 [48]. For sample digestion and other analytical procedures, all acids (including concentrated HNO_3_ and HCl) and reagents used were of analytical grade and supplied by MERCK (Darmstadt, Germany). Digestion was conducted under a laboratory chemical fume hood with controlled heating until the solution reached boiling and was maintained for 30 min. After cooling to an ambient laboratory temperature, the digested mixture was filtered to remove undissolved residues, and the filtrate was stored in clean 150 mL polyethylene bottles as stock solutions for subsequent analysis.

The total concentrations of Cd, Zn, Pb, and Mn were quantified using atomic absorption spectrometry (AAS) [49]. The measurements were performed with a PerkinElmer Analyst 200 instrument,(PerkinElmer, Shelton, CT, USA) operated using AA WinLab32 software version 6.5. Flame AAS (FAAS) was employed for atomization, utilizing a segmented solid-state detector with a double-beam optical system to ensure the high accuracy and reproducibility of metal quantification. As part of the quality assurance and quality control protocol, all calibration curves were rigorously evaluated for linearity, with R^2^ values required to exceed 0.995 to ensure a strong linear response across the working concentration range, with deionized water and standard solutions used for each calibration curve, along with the supplier (MERCK, Germany). Moreover, the limits of quantification (LOQs) were determined by analyzing multiple replicates of samples (3 times). Analysis of the samples alternates between the samples and the standard solution (known concentration) in order to ensure acceptable accuracy and precision.

#### 2.3.3. Determination of Heavy Metal Leachability

Three soil samples exhibiting significantly high concentrations of cadmium (Cd), lead (Pb), zinc (Zn), and manganese (Mn) were selected for heavy metal leachability testing. Each selected sample was subdivided into six sets (and each set has three replicates), with approximately 20 g of finely powdered soil (both 80-mesh and 200-mesh fractions) allocated per set. Each set was used to evaluate leachability under a distinct solution condition, simulating variations in natural environmental parameters.

The 20 g soil subsamples were placed into clean 150 mL polyethylene bottles, to which 120 mL of a test solution was added. These test solutions varied in pH and ionic strength to represent plausible environmental conditions that influence metal mobility. pH adjustments were made using diluted hydrochloric acid (HCl) and sodium hydroxide (NaOH), while ionic strength was controlled using sodium perchlorate (NaClO_4_), a non-reactive salt that does not interfere with other chemical species in solution. The six experimental conditions, combining different levels of pH and ionic strength, are summarized in Table 1. Sodium perchlorate was chosen as the electrolyte due to its chemical inertness and minimal complexation with metals, thereby minimizing interference with metal speciation during the experiment [50,51]. The selected ionic strengths and pH values reflect the soil solution conditions typically observed in contaminated agricultural environments and allow us to systematically assess their influence on metal mobility. These parameters are commonly employed in similar leaching studies to evaluate potential environmental risk and metal bioavailability [52,53].

Each soil–solution mixture was thoroughly agitated to ensure complete interaction between the soil particles and the test solution, facilitating the accurate assessment of metal leaching behavior under the specified conditions.

For each duration time, the resulting solution would be separated from 20 mL of solution using a centrifuge and by pipetting only clear solution. The centrifuge was used in each mixture for 3500 rounds per minute (rpm) for ten minutes. The heavy metal leachabilities in this study were sampled for five duration times, including 1 day, 3 days, 7 days, 14 days, and 28 days. After each duration of sampling, every mixture needed to be shaken for mixing again. The separated clear solutions for each duration were measured for the leached amount of cadmium, zinc, lead, and manganese using an atomic absorption spectrometer to determine the heavy metals’ leachability in the various solution conditions. Finally, the raw data was analyzed using a two-way ANOVA statistical method to identify whether the pH or ionic strength was a significant factor in heavy metal leaching and to determine the relationship between two variances.

### 2.4. Mineralogical Analysis Using X-Ray Diffractometer

In this step, the mineral composition of the powder soil samples in 200-mesh sieves, which are the same samples as in the heavy metal leaching test, were analyzed using BrukerD8 X-ray diffractometer (Bruker Corporation, Billerica, MA, USA), equipped with copper anode, at the Department of Geological Sciences, Faculty of Science, Chiang Mai University. X-ray diffraction (XRD) was performed on powder samples using an X-ray wavelength of 1.540598 nm. The identification and quantification of samples were assisted by EVA (search-match program) in the database of the International Centre for Diffraction Data (ICDD). The conditions of use were as follows: voltage 40 kV, stop 2θ 2 degrees, step size 0.04 degrees, time/step divergence 0.5 degrees, and anti-scattering slit.

### 2.5. Health Risk Assessment Method

In general, health risk assessment for metal-contaminated soils involves evaluating exposure through three primary pathways: ingestion, inhalation, and dermal absorption [54]. In this study, the assessment focused on the ingestion and dermal contact routes, as the inhalation pathway is considered to contribute minimally to heavy metal exposure in agricultural settings. The average daily dose (ADD) for both ingestion and dermal absorption was calculated following the guidelines provided by the United States Environmental Protection Agency (USEPA) [55]. The calculations were performed using the standard equations outlined by the USEPA, as shown in Equation (1) for ingestion and Equation (2) for dermal absorption, respectively.

(1) $$ADD_{\mathrm{ing}}=\frac{C\times I\times EF\times D}{A\times W},$$

where C is the concentration of heavy metals in soil (mg/kg) (data from laboratory analysis), I is the ingestion rate (100 mg/d for adults and 200 mg/d for children [56]), *EF* is the exposure frequency (350 days per year [56]), D is the exposure duration (70 years for adults and 6 years for children), W is the body weight (56.7 kg for adults [57] and 16.2 kg for children [54]), and A is average lifespan (70 × 350 d for adults and 6 × 350 d for children [56]).

(2) $$ADD_{\mathrm{derm}}=\frac{C\times ESA\times AF\times ABS\times EF\times D}{A\times W}$$

ESA is the exposed skin surface area (5700 cm^2^ for adults 2800 cm^2^ for children [58]), AF is the adherence factor (0.07 for adults 0.2 for children mg cm^−2^ [58]), and ABS is the dermal absorption factor (0.001 for all metals) [59,60].

It is important to note that soil exposure may occur through ingestion, dermal absorption, or a combination of both pathways. When heavy metal contamination was considered a non-carcinogenic health risk, the hazard quotient (HQ) was also evaluated using Equation (3) for ingestion and Equation (4) for dermal absorption [55]:

(3) $$HQ_{\mathrm{oral}}=\frac{ADD_{\mathrm{ing}}}{RfD_{o}},$$

(4) $$HQ_{\mathrm{abs}}=\frac{ADD_{\mathrm{derm}}}{RfD_{\mathrm{abs}}},$$

where $RfD_{o}$ is an oral references dose (1.00 × 10^−3^ mg/kg·d for Cd, 1.40 × 10^−3^ mg/kg·d for Pb, 3.00 × 10^−1^ mg/kg·d [61,62] for Zn, and 1.40 × 10^−1^ mg/kg·d for Mn [62]), RfD_abs_ is a dermal adsorption dose (1.00 × 10^−5^ mg/kg·d for Cd and 4.20 × 10^−4^ mg/kg·d for Pb [56,58,61,62]). To interpret the HQ, a value of the HQ greater than 1 indicates human health risk. The maximum acceptable carcinogenic risk is 1 × 10^−4^, which is considered an acceptable hazard [55].

It should be noted that carcinogenic health risk assessment only considered Cd and Pb as probable human carcinogens [56], not Zn or Mn, because the USEPA defined the Zn and Mn groups as not classifiable regarding human carcinogenicity [63,64]. Lifetime Cancer Risk (LCR) (Equations (5) and (6)) was analyzed for potential carcinogenic health risks [65].

(5) $$LCR_{\mathrm{oral}}=ADD_{\mathrm{ing}}\times CSF_{\mathrm{oral}},$$

(6) $$LCR_{\mathrm{derm}}=ADD_{\mathrm{derm}}\times CSF_{\mathrm{derm}},$$

where $CSF_{\mathrm{oral}}$ is the oral carcinogenic slope factor (6.1 mg/kg·d for Cd [66,67,68], 0.0085 mg/kg·d for Pb [66,69]). $CSF_{\mathrm{derm}}$ is the dermal carcinogenic slope factor (6.1 mg/kg·d for Cd [66], 0.0085 mg/kg·d for Pb [66,70]).

In addition, the total cancer risk from lifetime exposure (LCR) for Cd and Pb was estimated based on the accumulative values of all exposure pathways using Equation (7) [69]:

(7) $$LCR_{\mathrm{cancer}}=LCR_{\mathrm{ing}}+LCR_{\mathrm{derm}}$$

If the carcinogenic risk (LCR) value is less than 1 × 10^−6^, it is considered to pose no significant cancer risk. A LCR value between 1 × 10^−6^ and 1 × 10^−4^ is generally regarded as an acceptable or tolerable risk range for humans. However, if the LCR value exceeds 1 × 10^−4^, it indicates a high risk of cancer development and may warrant regulatory concern or remediation.

## 3. Results

### 3.1. Basic Soil Properties

Basic soil properties—including the temperature, hydrogen ion activity (pH), electrical conductivity (EC), and oxidation-reduction potential (ORP or Eh)—were measured at multiple time intervals: 0.5, 1.5, 4.0, 12, 24, 36, and 48 h. Table 2 presents the descriptive statistics (minimum, maximum, mean, median, standard deviation, and 95% confidence intervals) for these parameters across the 39 soil samples.

The pH values ranged from 5.75 to 8.86, indicating that the soil varied from weakly acidic to weakly alkaline conditions over the course of the two-day observation period. A general decreasing trend in pH was observed with increasing time, suggesting progressive stabilization. Specifically, the pH values at each time interval were as follows: 7.17–8.86 (0.5 h), 6.94–8.56 (1.5 h), 6.61–8.47 (4.0 h), 5.96–7.63 (12 h), 6.06–7.60 (24 h), 5.75–7.29 (36 h), and 6.09–7.20 (48 h). At equilibrium, soil samples tended toward a neutral pH, with both mean and median values falling within the range of 6.91 to 7.91.

Electrical conductivity (EC) exhibited a wide range across samples, from 2.60 to 932 µS/cm, with mean and median values ranging from 9.7 to 139 µS/cm. In contrast, the oxidation-reduction potential (ORP) values showed relatively narrow variation, ranging between 214 and 366 mV, reflecting stable redox conditions across the samples.

### 3.2. Metal Contents, Leachability Test and Mineralogical Contants

#### 3.2.1. Metal Concentrations

The total metal concentrations were determined using an acid digestion method, in which concentrated acids were applied to extract metals from soil samples prepared in both 80-mesh and 200-mesh fractions. The concentrations of four target heavy metals—cadmium (Cd), zinc (Zn), lead (Pb), and manganese (Mn)—were analyzed in soil samples collected from the Mae Tao watershed. A statistical summary of the metal concentrations, along with the relevant regulatory limits, was compiled based on data from 39 samples. Table 3 presents the descriptive statistics for each heavy metal, including the minimum, maximum, mean, median, and standard deviation.

The cadmium contents of 80-mesh sieved soil samples ranged from 6.59 to 135 mg/kg, and the average was 23.90 mg/kg. All the values had a lower cadmium content in 200-mesh sieved soil samples, ranging from 7.40 to 251 mg/kg, and the mean was 30.00 mg/kg. In the same way, the amounts of zinc, lead, and manganese in 80-mesh sieved samples are smaller than those in 200-mesh soil samples. The zinc contents of 80-mesh sieved soil samples ranged from 45.6 to 2756 mg/kg, and the average was 338 mg/kg, lower than in 200-mesh samples, whose contents ranged from 51.6 to 3269 mg/kg, with a mean value of 406 mg/kg. The lead contents of 80-mesh sieved soil samples ranged from 5.40 to 144 mg/kg, with an average of 38.9 mg/kg. In the case of 200-mesh-sized soil samples, the lead content ranged from 7.20 to 235 mg/kg, and the mean was 45.4 mg/kg. Moreover, in 80-mesh samples, the amount of manganese was found to range from approximately 139 to 2917 mg/kg, with a mean value of 892 mg/kg. The manganese contents ranged from 166 to 3255 mg/kg, with an average of 936 mg/kg in 200-mesh samples. This study showed that the larger soil samples (80-mesh) contained lower contents than the small, sieved soil samples (200-mesh) of all the types of heavy metals.

In addition, the comparison of the measured heavy metals with the standard values from the European Union (EU) [71] and the Pollution Control Department (PCD) maximum permissible [72] was considered, in order to carry out a preliminary risk assessment. All the statistical values of cadmium content were significantly greater than the maximum allowance, despite minimum values of Cd (6.59 mg/kg for 80-mesh and 7.40 mg/kg for 200-mesh) that exceeded approximately two times the guideline values (3 mg/kg), but all values of Pb were lower than the standard values (300 mg/kg). Considering Zn and Mn, the average values of Zn (338 mg/kg for 80-mesh and 406 mg/kg for 200-mesh) were over the European Union maximum permissible, which is 300 mg/kg, but Mn (892 mg/kg for 80-mesh and 936 mg/kg for 200-mesh) had acceptable values; however, the maximum values of Zn (2756 mg/kg for 80-mesh and 3269 mg/kg for 200-mesh) and Mn (2917 mg/kg for 80-mesh and 3255 mg/kg for 200-mesh) were above the EU maximum permissible by 10 and 1.6 times, respectively. Notably, the total concentrations of Cd, Zn, Pb, and Mn were 5952 mg/kg for 80-mesh and 7010 mg/kg for 200-mesh.

#### 3.2.2. Leachability of Metals

Three soil samples (S04, S12, and S19), exhibiting the highest total concentrations of cadmium (Cd), zinc (Zn), lead (Pb), and manganese (Mn), were selected for the leaching experiment. For each site, both 80-mesh and 200-mesh sieved fractions were tested, yielding a total of six experimental soil samples. The leachability of heavy metals was assessed under controlled laboratory conditions, by varying two key factors: ionic strength (0.01 M and 0.10 M NaClO_4_) and pH (adjusted to 4, 7, and 10). The leaching tests were conducted over a 28-day period, with leachate samples collected on days 1, 3, 7, 14, and 28. The concentrations of leached metals were quantified using atomic absorption spectrometry (AAS).

Overall, the leached concentrations were substantially lower than the corresponding total metal contents, and the leaching behavior was strongly influenced by both the soil particle size and ionic strength (see Tables S1–S4). The data also showed variability over time. To assess whether steady-state leaching was achieved, leached concentrations after 14 days were analyzed using linear regression. Following the criterion that a steady state is reached when the slope of the concentration-versus-time curve is not statistically different from zero [73], regression analysis (performed in Microsoft Excel^®^) confirmed that the leaching rates had stabilized by day 14 (*p* > 0.05); see Figures S1–S4.

The leaching percentages for Cd, Zn, Pb, and Mn were calculated as the ratio of metal leached at day 14 to the total metal content in each sample. These percentages were further analyzed using two-way ANOVA, with replications to evaluate the significance of pH and ionic strength effects. The results are summarized in Table 4. A statistically significant effect was indicated when the calculated *F*-value exceeded the *F*-critical value [74].

**Figure 2:**
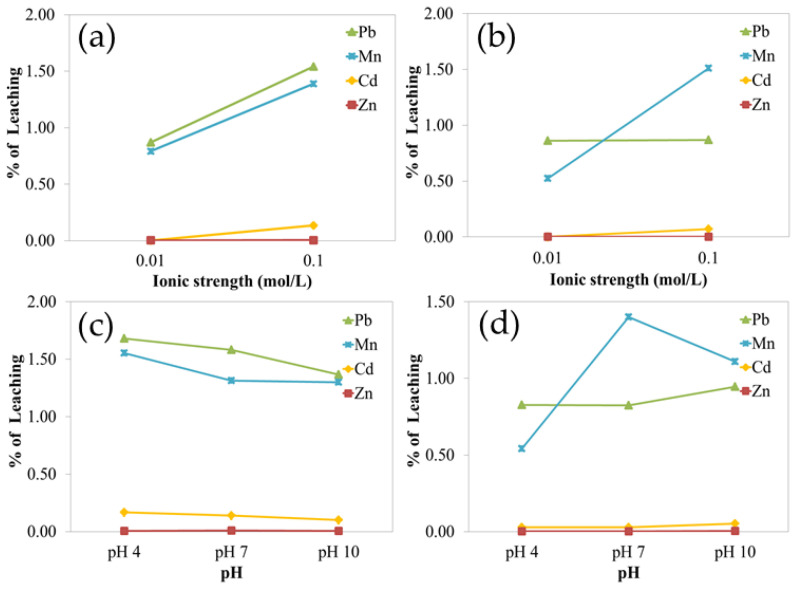
Graph summarizing two-way ANOVA results of the percentages of leaching of lead, manganese, cadmium, and zinc for ionic strength; (**a**) ionic strength factor of 80-mesh-sized samples, (**b**) ionic strength factor of 200-mesh-sized samples; (**c**) pH factor of 80-mesh-sized samples; and (**d**) pH factor of 200-mesh-sized samples.

The analysis revealed that ionic strength had a significant impact on Cd leaching in both 80-mesh and 200-mesh samples, and on Pb leaching in the 80-mesh fraction. In contrast, Zn and Mn leaching were not significantly affected by ionic strength. As shown in Figure 2, soils subjected to the higher ionic strength (0.10 M) exhibited markedly greater leaching of Cd and Pb compared to the 0.01 M treatment. This indicates enhanced metal mobility under higher ionic strength conditions.

Regarding pH, no statistically significant differences in leaching were observed across the pH levels tested. Furthermore, the metal leaching trends varied: Cd, Zn, and Pb showed relatively stable leaching profiles, while Mn (particularly in the 200-mesh samples) exhibited fluctuating concentrations over time (Figure 2), indicating an unstable leaching pattern.

#### 3.2.3. Mineralogical Composition of Soils

An X-ray diffraction technique was used to analyze the mineral compositions and carry out the semi-quantitative analysis, in which the results were applied in the stimulation part. This procedure used three 200-mesh sieved representatives. The XRD results are shown in Figure S3 and the semi-quantitative analysis of mineral compositions can be tentatively carried out using an X-ray diffractometer (XRD), using the percentage of all mineral compositions in the soil samples, as summarized in Table 5. The mineral compositions of samples mainly consisted of four minerals, namely quartz (76.65–87.91%), magnesium calcite (2.84–4.82%), dolomite (5.45–11.91%), and clay mineral (0–7.87%). Clay minerals disappeared from one out of three samples. The clay mineral contents of the two samples were from different categories. One sample found only illite (3.79%), and the other contained both illite (3.30%) and chlorite (4.57%). It should be noted that X-ray diffraction (XRD) was employed to identify the major mineralogical composition of soil samples. While XRD is effective for detecting dominant, well-crystallized phases, its utility is limited for trace-level or poorly crystalline constituents. The detection threshold for XRD typically exceeds 1–2 wt%, meaning that low-abundance Cd-bearing phases may not be observable. Furthermore, XRD does not provide information on the chemical speciation or binding state of trace metals such as cadmium. Therefore, the absence of identifiable Cd minerals in our analysis does not preclude the presence of Cd in labile or adsorbed forms. This limitation was considered in the interpretation of results and highlights the need for complementary techniques in future studies.

### 3.3. Health Risk Assessment Calculation

Non-carcinogenic risk was assessed through the calculation of the average daily dose (ADD, in mg/kg × d) and total hazard quotient (HQ) for four heavy metals: cadmium (Cd), lead (Pb), zinc (Zn), and manganese (Mn). These calculations were based on the mean, minimum, and maximum concentrations of each metal in two particle size fractions (80-mesh and 200-mesh) and considered two exposure pathways: ingestion and dermal contact. The ADD and HQ values were evaluated separately for adults and children, as summarized in Table 6 and Table 7, respectively.

The results indicated that the ADD values for ingestion ($ADD_{\mathrm{ing}}$) were significantly higher than those for dermal absorption ($ADD_{\mathrm{derm}}$) across all metals and age groups. The ADD values were comparable between adults and children, but ingestion remained the dominant exposure route. A positive correlation was observed between the metal concentration and ADD, with the ranking of average intake levels as follows: Mn > Zn > Cd > Pb.

In contrast, the hazard quotient (HQ) values followed a different trend: Cd > Pb > Zn > Mn. Notably, the HQ values for children were approximately one to two times higher than those for adults, reflecting their increased vulnerability. Additionally, the HQs were higher for the smaller particle size (200-mesh), indicating the greater bioavailability of metals in finer soils.

Despite these differences, all HQ values for both adults and children remained below the threshold of 1, suggesting no significant non-carcinogenic health risk. However, it is important to note that both adults and children are sensitive to the ingestion of Cd and Pb, while adults represent a more sensitive population group for the dermal adsorption of Cd. These results may require special attention in long-term exposure scenarios.

For the carcinogenic risk assessment (Table 8), the calculated cancer risk (CR) values for cadmium (Cd) indicate that exposure via ingestion at both the maximum and average values of concentrations in both 80-mesh and 200-mesh soil exceeds the acceptable threshold (CR > 1 × 10^−4^) for both adults and children, suggesting a potentially significant cancer risk from this pathway. In the case of dermal adsorption, the concentrations of cadmium and lead in both grain sizes resulted in a lifetime cancer risk (LCR) value of less than 1 × 10^−4^, indicating no carcinogenic concern via dermal exposure.

These findings suggest that elevated Cd concentrations in fine soil particles (200-mesh) pose a notable carcinogenic risk through ingestion for both adults and children, and through the ingestion of the average and maximum concentration levels. In contrast, the LCR values for lead (Pb) through both the oral and dermal exposure pathways ranged from 9.39 × 10^−10^ (dermal) to 1.24 × 10^−6^ (oral). These values fall within or below the generally accepted risk range of 1 × 10^−6^ to 1 × 10^−4^, with values below 1 × 10^−6^ considered to indicate no significant carcinogenic risk. Therefore, the carcinogenic risk associated with Pb exposure in this study is considered negligible to acceptable under the current exposure conditions.

## 4. Discussion

### 4.1. Basic Properties of Soils

The analysis of basic soil properties across samples collected from the Mae Tao watershed revealed dynamic changes in soil chemistry over time. In particular, the pH values of soil–water suspensions demonstrated a notable shift from weakly alkaline to neutral or slightly acidic conditions over a 48 h observation period. The initial alkaline conditions gradually declined with increasing time, suggesting the onset of chemical equilibration. This trend can be attributed to the activity of hydrogen (H^+^) and aluminum (Al^3+^) ions, which are key contributors to soil acidity. Aluminum ions undergo hydrolysis in the aqueous phase, forming AlOH^2+^ and releasing protons (H^+^), which in turn reduce pH levels over time [75,76]. Each Al^3+^ ion can produce up to three H^+^ ions, amplifying the acidifying effect in the soil solution during equilibration. Despite this acidification, the mean and median pH values across all samples remained within the neutral range of 6.91–7.91, indicating that the soils tend to stabilize around a neutral pH under natural conditions.

The electrical conductivity (EC) values varied widely among the samples, ranging from 2.60 to 932 µS/cm. This wide distribution indicates heterogeneity in the soluble salt content, likely influenced by agricultural practices such as the use of chemical fertilizers and irrigation water sourced from contaminated streams, particularly the Mae Tao Creek [77]. Over time, the EC values exhibited a consistent increase, driven by the dissolution of salts and ions present in the soil into the initially deionized water used for extraction. As equilibrium was approached, ion release stabilized, reflecting the chemical dynamics of soil–water interactions.

Oxidation-reduction potential (Eh) measurements showed moderate oxidation conditions across all samples, with values ranging between 214 and 366 mV. None of the samples exhibited reducing conditions (Eh < 100 mV). These values suggest that the soils are aerated and not waterlogged, which is consistent with agricultural usage. The combined data for pH, EC, and Eh further suggest that the soils exhibit considerable variability in physicochemical properties, likely due to the differential impacts of anthropogenic inputs, including fertilizers, pesticides, irrigation, and industrial discharges. This heterogeneity has important implications for the behavior, mobility, and bioavailability of heavy metals in the soil matrix.

### 4.2. Total Heavy Metal Concentrations and Soil Particle Size Effects

The total concentrations of cadmium (Cd), zinc (Zn), lead (Pb), and manganese (Mn) were determined for both 80-mesh and 200-mesh sieved soil fractions. Notably, the finer 200-mesh particles consistently exhibited higher concentrations of all four metals. For instance, the Cd levels in 200-mesh samples ranged up to 251 mg/kg (mean: 30.00 mg/kg), significantly higher than the 135 mg/kg maximum observed in 80-mesh samples (mean: 23.90 mg/kg). Similar trends were observed for Zn, Pb, and Mn. This size-dependent distribution reflects the greater specific surface area of finer particles, which enhances the sorption capacity and metal retention via surface complexation and ion exchange [76].

These findings confirm that particle size plays a critical role in determining the total metal concentrations in soil, and that finer particles present a greater risk to metal mobilization and bioavailability. The increased surface area in smaller particles provides more reactive sites for the adsorption of metals, making them more relevant when assessing environmental risk and remediation potential.

### 4.3. Comparison with Regulatory Standards

When compared with the regulatory limits established by the European Union (EU) [71] and Thailand’s Pollution Control Department (PCD) [72], the concentrations of Cd in all samples exceeded the permissible thresholds, with even the minimum values surpassing the guideline levels by a factor of two or more. The Zn and Mn levels were also elevated; the average Zn concentration exceeded the EU limits, and the maximum concentrations exceeded the permissible levels by 10-fold (Zn) and 1.6-fold (Mn). In contrast, the Pb concentrations remained below the regulatory thresholds in all cases.

These results indicate that the Mae Tao watershed is significantly contaminated with Cd, moderately contaminated with Zn and Mn, and not notably impacted by Pb. The elevated Cd levels raise concern due to its high toxicity, persistence, and potential for bioaccumulation in agricultural systems. The excessive Cd concentrations are consistent with previous studies linking contamination in this area to long-term zinc mining and industrial activity upstream of the Mae Tao Creek.

### 4.4. Heavy Metal Leachability

Leaching experiments were conducted on the three most contaminated soil samples (S04, S12, and S19) under varying pH and ionic strength conditions. Results showed that metal leachability was strongly influenced by ionic strength, especially for Cd and Pb. At a higher ionic strength (0.10 M), the electrical double layer surrounding soil particles is compressed, reducing electrostatic repulsion and increasing metal sorption, thus decreasing leachability [78,79,80]. Conversely, lower ionic strength (0.01 M) expands the double layer, allowing for the greater mobility and leaching of Cd and Pb into solution.

In contrast, the leachability of Zn and Mn was not significantly influenced by ionic strength, suggesting that their retention in soil is governed by other factors, such as specific adsorption to mineral surfaces or incorporation into secondary minerals. The influence of pH on leachability was not statistically significant in this study, although its role in surface protonation and the deprotonation of soil minerals is known to affect metal mobility under different conditions [81,82]. These results support the conclusion that ionic strength is a dominant factor in Cd and Pb leaching in the study soils, while pH plays a more complex and secondary role.

### 4.5. Mineralogical Evidence and Metal Speciation

X-ray diffraction (XRD) analysis was conducted on representative samples to evaluate mineralogical compositions. Major phases included quartz (76.65–87.91%), dolomite (5.45–11.91%), magnesium calcite (2.84–4.82%), and minor clay minerals (0–7.87%). Notably, no crystalline phases of cadmium-bearing minerals such as otavite (CdCO_3_) or greenockite (CdS) were detected, suggesting that Cd exists in the soil primarily in adsorbed or amorphous forms rather than as discrete mineral phases. This supports the interpretation that the observed Cd in leachates originates from loosely bound or exchangeable fractions rather than from the dissolution of cadmium minerals.

### 4.6. Health Risk Assessment

The human health risk assessment included an evaluation of both non-carcinogenic and carcinogenic risks via the ingestion and dermal exposure pathways. Non-carcinogenic risks were quantified using the hazard quotient (HQ) based on the average daily dose (ADD). The HQ values for all metals (Cd, Pb, Zn, and Mn) in both adults and children were below the threshold of 1.0, indicating no significant health risk under the current exposure conditions. Ingestion was the dominant exposure route, with the $ADD_{\mathrm{ing}}$ consistently higher than the $ADD_{\mathrm{derm}}$ across all metals. This aligns with the established exposure patterns for soil contaminants.

Despite earlier studies reporting adverse health outcomes in the local population and concerns about Cd exposure from rice consumption [27,31], the present assessment based on soil exposure pathways (ingestion and dermal contact) suggests that non-carcinogenic risk is minimal. One possible explanation for this is that the sampled soils, collected at a depth of 30 cm, may represent a leached zone with lower bioavailable concentrations of contaminants compared to surface soils, where human exposure is more direct.

However, the carcinogenic risk assessment revealed more concerning results. The lifetime cancer risk (LCR) values for Cd exceeded the critical threshold of 1 × 10^−4^ in some scenarios, particularly in ingestion pathways for both adults and children, using both 80-mesh and 200-mesh soil samples. This suggests that Cd in fine soil particles poses a potential carcinogenic risk, especially through the oral pathway. The dermal-based exposure LCR for Cd exceeded the acceptable limits all of concentrations in both 80-mesh and 200-mesh samples [55]. In contrast, Pb’s LCR values were within or below the acceptable range (1 × 10^−6^ to 1 × 10^−4^ and less than 1 × 10^−6^), indicating negligible carcinogenic risk.

Adults exhibited slightly higher vulnerability compared to children, with estimated hazard quotient (HQ) and lifetime cancer risk (LCR) values approximately one to two times greater across all exposure pathways. This increased susceptibility is primarily attributed to the longer lifespan of adults, which extends the duration of exposure and thereby amplifies both non-carcinogenic and carcinogenic risk accumulations over time, particularly from early life stages onward.

### 4.7. Implications and Recommendations

The results of this study highlight the complex interplay between soil properties, heavy metal contamination, particle size, leachability, and potential health risks. The Mae Tao watershed remains heavily impacted by cadmium contamination, with measurable risks for long-term human exposure, particularly through the oral pathway with both coarse and fine soil particles. While non-carcinogenic risk appears low under the current exposure assumptions, the carcinogenic potential of Cd warrants continued monitoring, the stricter regulation of agricultural land use, and targeted remediation efforts.

Future work should prioritize surface soil sampling (<15 cm depth), seasonal variations, and food chain transfer, particularly in rice and vegetable crops. Additional studies on metal speciation, bioavailability, and long-term soil–plant–human interactions are essential for developing a comprehensive risk management strategy for the region.

## 5. Conclusions

This study provides a comprehensive evaluation of heavy metal contamination, leachability behavior, and the associated human health risks in agricultural soils of the Mae Tao watershed, Mae Sot District, Tak Province, Thailand. The findings revealed elevated concentrations of cadmium (Cd), zinc (Zn), lead (Pb), and manganese (Mn) in both surface and subsurface soils, with Cd levels significantly exceeding the national and international regulatory thresholds [72]. While Zn and Mn were present at moderately elevated levels, Pb concentrations generally remained within the acceptable limits.

Leachability experiments demonstrated that ionic strength is a key factor influencing the mobility of heavy metals, particularly Cd and Pb, in the soil environment. Higher ionic strength was associated with increased metal desorption and mobility, thereby enhancing the potential for bioavailability and environmental transport. In contrast, pH showed no consistent or statistically significant effect on leaching behavior, likely due to its complex interactions with soil mineral surfaces and competing ions. These findings suggest that conventional assessments based solely on total metal concentrations may overestimate or underestimate actual environmental risk unless the influence of soil chemistry is properly accounted for.

Health risk assessments indicated that, under the current exposure scenarios via ingestion and dermal contact, none of the evaluated heavy metals posed a significant non-carcinogenic risk to either adults or children. However, carcinogenic risk from cadmium, particularly through ingestion exposure to both coarse (80-mesh) and fine soil particles (200-mesh), was found to exceed the acceptable threshold (1 × 10^−4^), signaling potential long-term health concerns. Lead also presented a minor carcinogenic risk, but remained within the acceptable limits.

Overall, this study underscores the importance of understanding the geochemical behavior of heavy metals in agricultural soils. Factors such as particle size, ionic strength, and mineralogical composition play critical roles in determining metal mobility and risk potential. The complex and dynamic nature of soil systems necessitates an integrated approach for effective environmental monitoring and risk management in contaminated agricultural landscapes.

## Figures and Tables

**Table 1:** The conditions of solution in leaching test.

Condition	pH	Ionic Strength (mol/L)
1	4.0	0.01
2	7.0	0.01
3	10.0	0.01
4	4.0	0.1
5	7.0	0.1
6	10.0	0.1

**Table 2:** The values of pH, EC and ORP of soils measured at different times.

Variable		Min	Max	Mean	Median	SD	95% IC
pH	0.5 h	7.17	8.86	7.96	7.79	0.53	0.17
	1.5 h	6.94	8.56	7.89	7.93	0.39	0.12
	4 h	6.61	8.47	7.79	7.79	0.36	0.11
	12 h	5.96	7.63	6.98	7.09	0.44	0.14
	24 h	6.06	7.60	6.95	6.95	0.41	0.13
	36 h	5.75	7.29	6.71	6.79	0.37	0.12
EC (μS/cm)	0.5 h	2.60	194	21.4	9.7	36	11.4
	1.5 h	3.90	268	34.0	17.3	55.5	17.6
	4 h	5.40	320	44.7	28.0	66.4	21.1
	12 h	6.50	588	78.8	43.5	125	39.8
	24 h	11.10	755	110	76.2	158	50.1
	36 h	11.80	817	126	83.1	183	58.3
	48 h	13.00	932	139	90.2	204	64.7
ORP (mV)	0.5 h	123	299	217	223	40.0	12.6
	1.5 h	138	329	223	224	43.1	13.6
	4 h	160	337	241	241	33.8	10.7
	12 h	168	319	214	210	36.1	11.4
	24 h	206	366	244	237	35.3	11.3
	36 h	209	306	247	243	24.5	7.73
	48 h	214	342	262	262	25.3	7.99

**Table 3:** Heavy metal contents in soils.

Metals	Size	Min	Max	Mean	Median	Regulated Standard
Cd (mg/kg)	80-mesh	6.59	135	23.90	17.40	3 *
	200-mesh	7.40	251	30.00	17.80	3 *
Zn (mg/kg)	80-mesh	45.60	2756	338	128	300 *
	200-mesh	51.70	3269	406	147	300 *
Pb (mg/kg)	80-mesh	5.40	144	38.90	31.40	300 *
	200-mesh	7.20	235	45.40	33.40	300 *
Mn (mg/kg)	80-mesh	139	2917	892	792	1800 **
	200-mesh	166	3255	936	810	1800 **

Sources: European Union (EU) maximum permissible [71] *, the Pollution Control Department (PCD) maximum permissible [72] **.

**Table 4:** Two-way ANOVA results of cadmium, zinc, lead, and manganese in both 80- and 200-mesh-sized samples.

Cd	80-Mesh					200-Mesh				
Variation	Sum of Squares	Mean Square	F	*p*-Value	F-Crit	Sum of Squares	Mean Square	F	*p*-Value	F-Crit
Ionic strength	0.083	0.084	71.710	0.000	4.747	0.023	0.023	21.592	0.001	4.747
pH	0.004	0.002	1.525	0.257	3.885	0.002	0.001	1.026	0.388	3.885
Interaction	0.004	0.002	1.525	0.257	3.885	0.002	0.001	1.026	0.388	3.885
Within	0.014	0.001				0.013	0.001			
Total	0.105					0.040				
**Zn**	80-mesh					200-mesh				
Ionic strength	1.27 × 10^−6^	1.27 × 10^−6^	0.048	0.830	4.747	4.67 × 10^−8^	4.67 × 10^−8^	0.0073	0.933	4.747
pH	2.52 × 10^−5^	1.26 × 10^−5^	0.475	0.633	3.885	2.73 × 10^−5^	1.37 × 10^−5^	2.1251	0.162	3.885
Interaction	2.57 × 10^−5^	1.28 × 10^−5^	0.485	0.627	3.885	1.42 × 10^−5^	7.11 × 10^−6^	1.1053	0.363	3.885
Within	3.18 × 10^−4^	2.65 × 10^−5^				7.72 × 10^−5^	6.43 × 10^−6^			
Total	3.70 × 10^−4^					1.19 × 10^−4^				
**Pb**	80-mesh					200-mesh				
Ionic strength	2.028	2.028	4.966	0.046	4.747	0.000	0.000	0.000	0.985	4.747
pH	0.409	0.205	0.501	0.618	3.885	0.058	0.029	0.119	0.889	3.885
Interaction	0.094	0.047	0.115	0.892	3.885	0.328	0.164	0.680	0.525	3.885
Within	4.902	0.409				2.891	0.241			
Total	7.434					3.276				
**Mn**	80-mesh					200-mesh				
Ionic strength	1.604	1.604	1.383	0.262	4.747	4.380	4.380	3.425	0.089	4.747
pH	0.019	0.009	0.008	0.992	3.885	2.286	1.143	0.894	0.435	3.885
Interaction	0.287	0.143	0.124	0.885	3.885	0.946	0.473	0.370	0.699	3.885
Within	13.917	1.160				15.350	1.279			
Total	15.826					22.962				

**Table 5:** Results of semi-quantitative analysis of soil samples using XRD.

Sample No. (Name)	Composition				
	Quartz (%)	Magnesium Calcite (%)	Dolomite (%)	Clay Minerals	
				Illite (%)	Chlorite (%)
1 (S4)	84.62	3.47	11.91	-	-
2 (S12)	87.91	2.84	5.45	3.79	-
3 (S19)	76.65	4.82	10.66	3.30	4.57

**Table 6:** Average daily dose (ADD) and hazard quotient (HQ) of heavy metals via ingestion and dermal adsorption from agricultural soils for adults.

Metals	Value	Size	Concentration (mg/kg)	${\mathrm{ADD}}_{\mathrm{ing}}$	${\mathrm{ADD}}_{\mathrm{derm}}$	${\mathrm{HQ}}_{\mathrm{ing}}$	${\mathrm{HQ}}_{\mathrm{derm}}$
Cd	Mean	80-mesh	23.90	4.04 × 10^−5^	1.61 × 10^−7^	4.04 × 10^−2^	1.61 × 10^−2^
		200-mesh	30.00	5.07 × 10^−5^	2.02 × 10^−7^	5.07 × 10^−2^	2.02 × 10^−2^
	Min	80-mesh	6.59	1.11 × 10^−5^	4.45 × 10^−8^	1.11 × 10^−2^	4.45 × 10^−3^
		200-mesh	7.40	1.25 × 10^−5^	4.99 × 10^−8^	1.25 × 10^−2^	4.99 × 10^−3^
	Max	80-mesh	135.00	2.28 × 10^−4^	9.11 × 10^−7^	2.28 × 10^−1^	9.11 × 10^−2^
		200-mesh	251.00	4.24 × 10^−4^	1.69 × 10^−6^	4.24 × 10^−1^	1.69 × 10^−1^
Pb	Mean	80-mesh	38.90	6.58 × 10^−5^	2.62 × 10^−7^	4.70 × 10^−2^	6.25 × 10^−4^
		200-mesh	45.40	7.68 × 10^−5^	3.06 × 10^−7^	5.48 × 10^−2^	7.29 × 10^−4^
	Min	80-mesh	5.40	9.13 × 10^−6^	3.64 × 10^−8^	6.52 × 10^−3^	8.68 × 10^−5^
		200-mesh	7.20	1.22 × 10^−5^	4.86 × 10^−8^	8.70 × 10^−3^	1.16 × 10^−4^
	Max	80-mesh	144.00	2.44 × 10^−4^	9.72 × 10^−7^	1.74 × 10^−1^	2.31 × 10^−3^
		200-mesh	235.00	3.97 × 10^−4^	1.59 × 10^−6^	2.84 × 10^−1^	3.78 × 10^−3^
Zn	Mean	80-mesh	338.00	5.72 × 10^−4^	2.28 × 10^−6^	1.91 × 10^−3^	-
		200-mesh	406.00	6.87 × 10^−4^	2.74 × 10^−6^	2.29 × 10^−3^	-
	Min	80-mesh	45.60	7.71 × 10^−5^	3.08 × 10^−7^	2.57 × 10^−4^	-
		200-mesh	51.70	8.74 × 10^−5^	3.49 × 10^−7^	2.91 × 10^−4^	-
	Max	80-mesh	2756.00	4.66 × 10^−3^	1.86 × 10^−5^	1.55 × 10^−2^	-
		200-mesh	3269.00	5.53 × 10^−3^	2.21 × 10^−5^	1.84 × 10^−2^	-
Mn	Mean	80-mesh	892.00	1.51 × 10^−3^	6.02 × 10^−6^	1.08 × 10^−2^	-
		200-mesh	936.00	1.58 × 10^−3^	6.32 × 10^−6^	1.13 × 10^−2^	-
	Min	80-mesh	139.00	2.35 × 10^−4^	9.38 × 10^−7^	1.68 × 10^−3^	-
		200-mesh	166.00	2.81 × 10^−4^	1.12 × 10^−6^	2.01 × 10^−3^	-
	Max	80-mesh	2917.00	4.93 × 10^−3^	1.97 × 10^−5^	3.52 × 10^−2^	-
		200-mesh	3255.00	5.50 × 10^−3^	2.20 × 10^−5^	3.93 × 10^−2^	-

**Table 7:** Average daily dose (ADD) and hazard quotient (HQ) of heavy metals via ingestion and dermal adsorption from agricultural soils for children.

Metals	Value	Size	Concentration (mg/kg)	${\mathrm{ADD}}_{\mathrm{ing}}$	${\mathrm{ADD}}_{\mathrm{derm}}$	${\mathrm{HQ}}_{\mathrm{ing}}$	${\mathrm{HQ}}_{\mathrm{derm}}$
Cd	Mean	80-mesh	23.90	2.43 × 10^−5^	6.79 × 10^−8^	2.43 × 10^−2^	6.79 × 10^−3^
		200-mesh	30.00	3.04 × 10^−5^	8.52 × 10^−8^	3.04 × 10^−2^	8.52 × 10^−3^
	Min	80-mesh	6.59	6.69 × 10^−6^	1.87 × 10^−8^	6.69 × 10^−3^	1.87 × 10^−3^
		200-mesh	7.40	7.51 × 10^−6^	2.10 × 10^−8^	7.51 × 10^−3^	2.10 × 10^−3^
	Max	80-mesh	135.00	1.37 × 10^−4^	3.84 × 10^−7^	1.37 × 10^−1^	3.84 × 10^−2^
		200-mesh	251.00	2.55 × 10^−4^	7.13 × 10^−7^	2.55 × 10^−1^	7.13 × 10^−2^
Pb	Mean	80-mesh	38.90	3.95 × 10^−5^	1.11 × 10^−7^	2.82 × 10^−2^	2.63 × 10^−4^
		200-mesh	45.40	4.61 × 10^−5^	1.29 × 10^−7^	3.29 × 10^−2^	3.07 × 10^−4^
	Min	80-mesh	5.40	5.48 × 10^−6^	1.53 × 10^−8^	3.91 × 10^−3^	3.65 × 10^−5^
		200-mesh	7.20	7.31 × 10^−6^	2.05 × 10^−8^	5.22 × 10^−3^	4.87 × 10^−5^
	Max	80-mesh	144.00	1.46 × 10^−4^	4.09 × 10^−7^	1.04 × 10^−1^	9.74 × 10^−4^
		200-mesh	235.00	2.38 × 10^−4^	6.68 × 10^−7^	1.70 × 10^−1^	1.59 × 10^−3^
Zn	Mean	80-mesh	338.00	3.43 × 10^−4^	9.60 × 10^−7^	1.14 × 10^−3^	-
		200-mesh	406.00	4.12 × 10^−4^	1.15 × 10^−6^	1.37 × 10^−3^	-
	Min	80-mesh	45.60	4.63 × 10^−5^	1.30 × 10^−7^	1.54 × 10^−4^	-
		200-mesh	51.70	5.25 × 10^−5^	1.47 × 10^−7^	1.75 × 10^−4^	-
	Max	80-mesh	2756.00	2.80 × 10^−3^	7.83 × 10^−6^	9.32 × 10^−3^	-
		200-mesh	3269.00	3.32 × 10^−3^	9.29 × 10^−6^	1.11 × 10^−2^	-
Mn	Mean	80-mesh	892.00	9.05 × 10^−4^	2.53 × 10^−6^	6.47 × 10^−3^	-
		200-mesh	936.00	9.50 × 10^−4^	2.66 × 10^−6^	6.78 × 10^−3^	-
	Min	80-mesh	139.00	1.41 × 10^−4^	3.95 × 10^−7^	1.01 × 10^−3^	-
		200-mesh	166.00	1.68 × 10^−4^	4.72 × 10^−7^	1.20 × 10^−3^	-
	Max	80-mesh	2917.00	2.96 × 10^−3^	8.29 × 10^−6^	2.11 × 10^−2^	-
		200-mesh	3255.00	3.30 × 10^−3^	9.25 × 10^−6^	2.36 × 10^−2^	-

**Table 8:** Cancer risk of assessed metals via ingestion and dermal adsorption from agricultural soils.

Metals	Value	Age	Size	Concentration (mg/kg)	${\mathrm{LCR}}_{\mathrm{ing}}$	${\mathrm{LCR}}_{\mathrm{derm}}$	${\mathrm{LCR}}_{\mathrm{cancer}}$
Cd	Mean	Adults	80-mesh	23.90	2.47 × 10^−4^	9.84 × 10^−7^	2.48 × 10^−4^
			200-mesh	30.00	3.09 × 10^−4^	1.23 × 10^−6^	3.11 × 10^−4^
		Children	80-mesh	23.90	1.48 × 10^−4^	4.14 × 10^−7^	1.48 × 10^−4^
			200-mesh	30.00	1.86 × 10^−4^	5.20 × 10^−7^	1.86 × 10^−4^
	Min	Adults	80-mesh	6.59	6.80 × 10^−5^	2.71 × 10^−7^	6.83 × 10^−5^
			200-mesh	7.40	7.63 × 10^−5^	3.05 × 10^−7^	7.66 × 10^−5^
		Children	80-mesh	6.59	4.08 × 10^−5^	1.14 × 10^−7^	4.09 × 10^−5^
			200-mesh	7.40	4.58 × 10^−5^	1.28 × 10^−7^	4.59 × 10^−5^
	Max	Adults	80-mesh	135.00	1.39 × 10^−3^	5.56 × 10^−6^	1.40 × 10^−3^
			200-mesh	251.00	2.59 × 10^−3^	1.03 × 10^−5^	2.60 × 10^−3^
		Children	80-mesh	135.00	8.36 × 10^−4^	2.34 × 10^−6^	8.38 × 10^−4^
			200-mesh	251.00	1.55 × 10^−3^	4.35 × 10^−6^	1.56 × 10^−3^
Pb	Mean	Adults	80-mesh	38.90	5.59 × 10^−7^	2.23 × 10^−9^	5.61 × 10^−7^
			200-mesh	45.40	6.53 × 10^−7^	2.60 × 10^−9^	6.55 × 10^−7^
		Children	80-mesh	38.90	3.36 × 10^−7^	9.39 × 10^−10^	3.36 × 10^−7^
			200-mesh	45.40	3.92 × 10^−7^	1.10 × 10^−9^	3.93 × 10^−7^
	Min	Adults	80-mesh	5.40	7.76 × 10^−8^	3.10 × 10^−10^	7.79 × 10^−8^
			200-mesh	7.20	1.04 × 10^−7^	4.13 × 10^−10^	1.04 × 10^−7^
		Children	80-mesh	5.40	4.66 × 10^−8^	1.30 × 10^−10^	4.67 × 10^−8^
			200-mesh	7.20	6.21 × 10^−8^	1.74 × 10^−10^	6.23 × 10^−8^
	Max	Adults	80-mesh	144.00	2.07 × 10^−6^	8.26 × 10^−9^	2.08 × 10^−6^
			200-mesh	235.00	3.38 × 10^−6^	1.35 × 10^−8^	3.39 × 10^−6^
		Children	80-mesh	144.00	1.24 × 10^−6^	3.48 × 10^−9^	1.25 × 10^−6^
			200-mesh	235.00	2.03 × 10^−6^	5.68 × 10^−9^	2.03 × 10^−6^

## Data Availability

The groundwater flow model simulation input/output files, groundwater level measurements, and stable isotopic data are available upon request.

## Supplementary Materials

The following supporting information can be downloaded at: https://www.mdpi.com/article/10.3390/toxics13080687/s1. Table S1: Cadmium leachability test results; Table S2: Zinc leachability test results; Table S3: Lead leachability test results; Table S4: Manganese leachability test results; Figure S1: Percent leached concentrations of cadmium from soil samples number S04, S12, and S19 in 80-mesh and 200-mesh size in test time of 28 days; Figure S2: Percent leached concentrations of zinc from soil samples number S04, S12, and S19 in 80-mesh and 200-mesh size in test time of 28 days; Figure S3: Percent leached concentrations of lead from soil samples number S04, S12, and S19 in 80-mesh and 200-mesh size in test time of 28 days; and Figure S4: Percent leached concentrations of manganese from soil samples number S04, S12, and S19 in 80-mesh and 200-mesh size in test time of 28 day.
