# Supplementary material for: Leachability and Health Risk Assessment of Cadmium and Other Heavy Metals in Agricultural Soils from the Mae Tao Watershed, Northern Thailand
Source: Toxics. 2025 Aug 18;13(8):687. doi: 10.3390/toxics13080687 (PMC12390624; doi:10.3390/toxics13080687)
Supplement: Supplementary file 1 [file toxics-13-00687-s001.zip › toxics-3774190-supplementary.pdf]

Supplements

Table S1: Cadmium leachability test results.

Cadmium (Cd)									
Sample Number	Sieved Size (mesh)	Ionic Strength	pH	Soil Weight (grams)	Leached Concentration from AAS (mg/L)				
					Day 1	Day 3	Day 7	Day 14	Day 28
S04	80	0.1	4	20.0000	0.0354	0.0335	0.0373	0.0392	0.0373
			7	20.0022	0.0297	0.0297	0.0316	0.0297	0.0316
			10	20.0045	0.0354	0.0297	0.0335	0.0316	0.0316
		0.01	4	19.9972	0	0	0	0	0
			7	20.0004	0	0	0	0	
			10	20.0057	0	0	0	0	
	200	0.1	4	20.0028	0.0297	0.0297	0.0316	0.0278	
			7	20.0029	0.0278	0.0297	0.0278	0.0335	
			10	20.0026	0.0373	0.0297	0.0335	0.0392	0.0373
		0.01	4	20.0033	0	0	0	0	0
			7	20.0030	0	0	0	0	0
			10	20.0084	0	0	0	0	0
S12	80	0.1	4	20.0057	0.0335	0.0335	0.0335	0.0354	0.0392
			7	20.0049	0.0335	0.0316	0.0297	0.0278	0.0278
			10	19.9991	0.0335	0.0278	0.0316	0.0373	0.0335
		0.01	4	20.0048	0	0	0	0	0
			7	20.0023	0	0	0	0	0
			10	20.0061	0	0	0	0	0
	200	0.1	4	20.0093	0.0373	0.0411	0.0316	0.0354	0.0335
			7	19.9979	0.0354	0.0335	0.0316	0.0335	0.0316
			10	19.9995	0.0392	0.0316	0.0354	0.0354	0.0373
		0.01	4	19.9996	0	0	0	0	0
			7	20.0016	0	0	0	0	0
			10	20.0040	0	0	0	0	0
S19	80	0.1	4	20.0018	0.0411	0.0392	0.0354	0.0373	0.0392
			7	20.0074	0.0392	0.0411	0.0373	0.0392	0.0354
			10	20.0040	0.0354	0.0354	0.0392	0.0373	0.0392
		0.01	4	20.0020	0	0	0	0	0
			7	20.0013	0	0	0	0	0
			10	19.9988	0	0	0	0	0
	200	0.1	4	20.0011	0.0392	0.0411	0.0373	0.0392	0.0373
			7	20.0064	0.0411	0.0392	0.0430	0.0392	0.0373
			10	20.0020	0.0392	0.0449	0.0430	0.0411	0.0354
		0.01	4	20.0010	0	0	0	0	0
			7	20.0060	0	0	0	0	0
			10	19.9991	0	0	0	0	0

Table S2: Zinc leachability test results.

Zinc (Zn)									
Sample Number	Sieved Size (mesh)	Ionic Strength	pH	Soil Weight (grams)	Leached Concentration from AAS (mg/L)				
					Day 1	Day 3	Day 7	Day 14	Day 28
S04	80	0.1	4	20.0000	0.0994	0.1241	0.1282	0.1674	0.0416
			7	20.0022	0.0870	0.1035	0.1138	0.0932	0.0458
			10	20.0045	0.0643	0.1014	0.1324	0.0726	0.0416
		0.01	4	19.9972	0.0293	0.0561	0.0437	0.0396	0.0272
			7	20.0004	0.0334	0.0520	0.0375	0.0396	
			10	20.0057	0.0437	0.0252	0.0272	0.0293	
	200	0.1	4	20.0028	0.1262	0.1468	0.1571	0.0396	
			7	20.0029	0.1097	0.1447	0.1468	0.1159	
			10	20.0026	0.1097	0.1076	0.0561	0.0953	0.0602
		0.01	4	20.0033	0.0540	0.0685	0.0293	0.0210	0.0148
			7	20.0030	0.0478	0.0334	0.0334	0.0355	0.0128
			10	20.0084	0.0396	0.0746	0.0416	0.0272	0.0231
S12	80	0.1	4	20.0057	0.0932	0.1427	0.1427	0.1551	0.0829
			7	20.0049	0.0932	0.1138	0.1489	0.0623	0.2004
			10	19.9991	0.0808	0.1159	0.1427	0.1241	0.0623
		0.01	4	20.0048	0.0623	0.0458	0.0416	0.0396	0.0313
			7	20.0023	0.0396	0.0561	0.0499	0.0540	0.0416
			10	20.0061	0.0540	0.0499	0.0458	0.0499	0.1200
	200	0.1	4	20.0093	0.1097	0.1715	0.1757	0.1468	0.0375
			7	19.9979	0.1221	0.1489	0.1571	0.0870	0.0375
			10	19.9995	0.1138	0.1551	0.1489	0.1138	0.0499
		0.01	4	19.9996	0.0540	0.0788	0.0499	0.0355	0.0231
			7	20.0016	0.0458	0.0623	0.0458	0.0581	0.0169
			10	20.0040	0.0581	0.0643	0.0416	0.0355	0.0210
S19	80	0.1	4	20.0018	0.0788	0.1035	0.0973	0.0973	0.0767
			7	20.0074	0.0746	0.0932	0.1035	0.1035	0.0561
			10	20.0040	0.0664	0.0891	0.1014	0.0416	0.0849
		0.01	4	20.0020	0.0313	0.0148	0.0478	0.0313	0.0355
			7	20.0013	0.0272	0.0355	0.0355	0.0313	0.1014
			10	19.9988	0.0252	0.0396	0.0293	0.0313	0.1262
	200	0.1	4	20.0011	0.1262	0.0416	0.0849	0.0767	0.0437
			7	20.0064	0.1241	0.1200	0.0849	0.0788	0.0355
			10	20.0020	0.0478	0.1447	0.1159	0.0849	0.0726
		0.01	4	20.0010	0.0355	0.0561	0.0272	0.0190	0.0128
			7	20.0060	0.0396	0.0478	0.0334	0.0252	0.0128
			10	19.9991	0.0272	0.0499	0.0231	0.0252	0.0148

Table S3: Lead leachability test results.

Lead (Pb)									
Sample Number	Sieved Size (mesh)	Ionic Strength	pH	Soil Weight (grams)	Leached Concentration from AAS (mg/L)				
					Day 1	Day 3	Day 7	Day 14	Day 28
S04	80	0.1	4	20.0000	0.3020	0.3020	0.3837	0.3429	0.4245
			7	20.0022	0.3020	0.3837	0.3837	0.3429	0.3429
			10	20.0045	0.3020	0.3429	0.3020	0.3429	0.4245
		0.01	4	19.9972	0.1796	0.2204	0.1796	0.2204	0.2612
			7	20.0004	0.1388	0.2204	0.2204	0.2612	
			10	20.0057	0.1388	0.2204	0.2204	0.2612	
	200	0.1	4	20.0028	0.3429	0.3429	0.3429	0.3837	
			7	20.0029	0.2612	0.3837	0.3020	0.3429	
			10	20.0026	0.3837	0.3429	0.3837	0.3429	0.3020
		0.01	4	20.0033	0.1796	0.2612	0.2612	0.2612	0.2612
			7	20.0030	0.2204	0.2612	0.1796	0.3429	0.2204
			10	20.0084	0.1796	0.2612	0.2204	0.2612	0.2204
S12	80	0.1	4	20.0057	0.2612	0.3429	0.3429	0.4245	0.3429
			7	20.0049	0.2612	0.3429	0.3429	0.3837	0.4245
			10	19.9991	0.3020	0.3429	0.3429	0.4245	0.3429
		0.01	4	20.0048	0.1796	0.1796	0.1796	0.3020	0.2204
			7	20.0023	0.1796	0.2204	0.2612	0.2204	0.2612
			10	20.0061	0.2204	0.2204	0.2204	0.2612	0.3020
	200	0.1	4	20.0093	0.3429	0.3837	0.3020	0.3837	0.3429
			7	19.9979	0.3429	0.3837	0.3429	0.3429	0.3837
			10	19.9995	0.3429	0.3837	0.3837	0.4245	0.3429
		0.01	4	19.9996	0.2612	0.2204	0.2612	0.2204	0.3020
			7	20.0016	0.2204	0.2204	0.3020	0.2204	0.2612
			10	20.0040	0.2204	0.2612	0.2612	0.2612	0.2204
S19	80	0.1	4	20.0018	0.3020	0.3837	0.3429	0.3429	0.3837
			7	20.0074	0.3020	0.3020	0.3429	0.3429	0.3429
			10	20.0040	0.3020	0.3837	0.3429	0.3429	0.4245
		0.01	4	20.0020	0.2204	0.2612	0.2204	0.2204	0.3020
			7	20.0013	0.2204	0.2204	0.3020	0.2204	0.2612
			10	19.9988	0.1796	0.2204	0.3020	0.2612	0.2612
	200	0.1	4	20.0011	0.3837	0.3020	0.4245	0.4245	0.4245
			7	20.0064	0.3429	0.3837	0.3837	0.4245	0.3837
			10	20.0020	0.3429	0.3020	0.3837	0.3837	0.4245
		0.01	4	20.0010	0.2204	0.1796	0.3020	0.2612	0.3020
			7	20.0060	0.3020	0.2204	0.2204	0.2204	0.3837
			10	19.9991	0.2612	0.2204	0.3020	0.2612	0.2612

Table S4: Manganese leachability test results.

Manganese (Mn)									
Sample Number	Sieved Size (mesh)	Ionic Strength	pH	Soil Weight (grams)	Leached Concentration from AAS (mg/L)				
					Day 1	Day 3	Day 7	Day 14	Day 28
S04	80	0.1	4	20.0000	0.3482	0.6436	1.2925	1.7913	1.2877
			7	20.0022	0.3094	0.5031	1.1424	1.5298	1.6169
			10	20.0045	0.2562	0.5322	1.1569	1.4378	0.9341
		0.01	4	19.9972	0	0.3579	0.5855	0.7065	0
			7	20.0004	0	0.3337	0.4499	0.9971	
			10	20.0057	0.0722	0	0.3530	1.0019	
	200	0.1	4	20.0028	0.4838	0.9002	2.0576	0.5274	
			7	20.0029	0.4354	0.8082	1.9220	2.5370	
			10	20.0026	0.4015	0.7453	1.6363	2.5128	1.4765
		0.01	4	20.0033	0.0722	0.4257	0.4886	0.0528	0
			7	20.0030	0.1496	0.4402	0.6969	1.3458	0
			10	20.0084	0	0.3482	0.6048	0.3337	0.1400
S12	80	0.1	4	20.0057	0.0722	0.3191	0.7840	1.3119	1.8058
			7	20.0049	0.0673	0.2465	0.7308	1.0746	1.4039
			10	19.9991	0.0576	0.3288	0.7356	1.1956	1.5395
		0.01	4	20.0048	0	0.0576	0.2755	0.4741	0.4063
			7	20.0023	0	0.0915	0.6775	0.9196	0.3143
			10	20.0061	0	0.1061	0.3918	0.5709	0.3191
	200	0.1	4	20.0093	0.0576	0.3772	1.1956	2.1303	0.8567
			7	19.9979	0.0722	0.3385	1.2247	1.9511	0.9148
			10	19.9995	0.0770	0.4111	1.4523	2.5806	1.6315
		0.01	4	19.9996	0	0.2707	0.9002	0.9923	0.0770
			7	20.0016	0	0.2271	0.6097	1.4087	0
			10	20.0040	0	0.0673	0.7404	0.5177	0
S19	80	0.1	4	20.0018	0.0576	0.1206	0.1690	0.1400	0
			7	20.0074	0.0528	0.1012	0.1593	0.1496	0
			10	20.0040	0.0625	0.0915	0.1545	0.0915	0
		0.01	4	20.0020	0	0	0.1884	0.1157	0
			7	20.0013	0	0	0.1690	0.1642	0
			10	19.9988	0	0	0.1206	0.1303	0
	200	0.1	4	20.0011	0.1254	0.1835	0.2223	0.3676	0.1448
			7	20.0064	0.0818	0.1545	0.2804	0.6000	0.1351
			10	20.0020	0.0673	0.1932	0.2320	0.5225	0.1738
		0.01	4	20.0010	0	0.0867	0.2998	0.0528	0
			7	20.0060	0	0	0.3482	0.0867	0
			10	19.9991	0	0	0.3143	0.0964	0

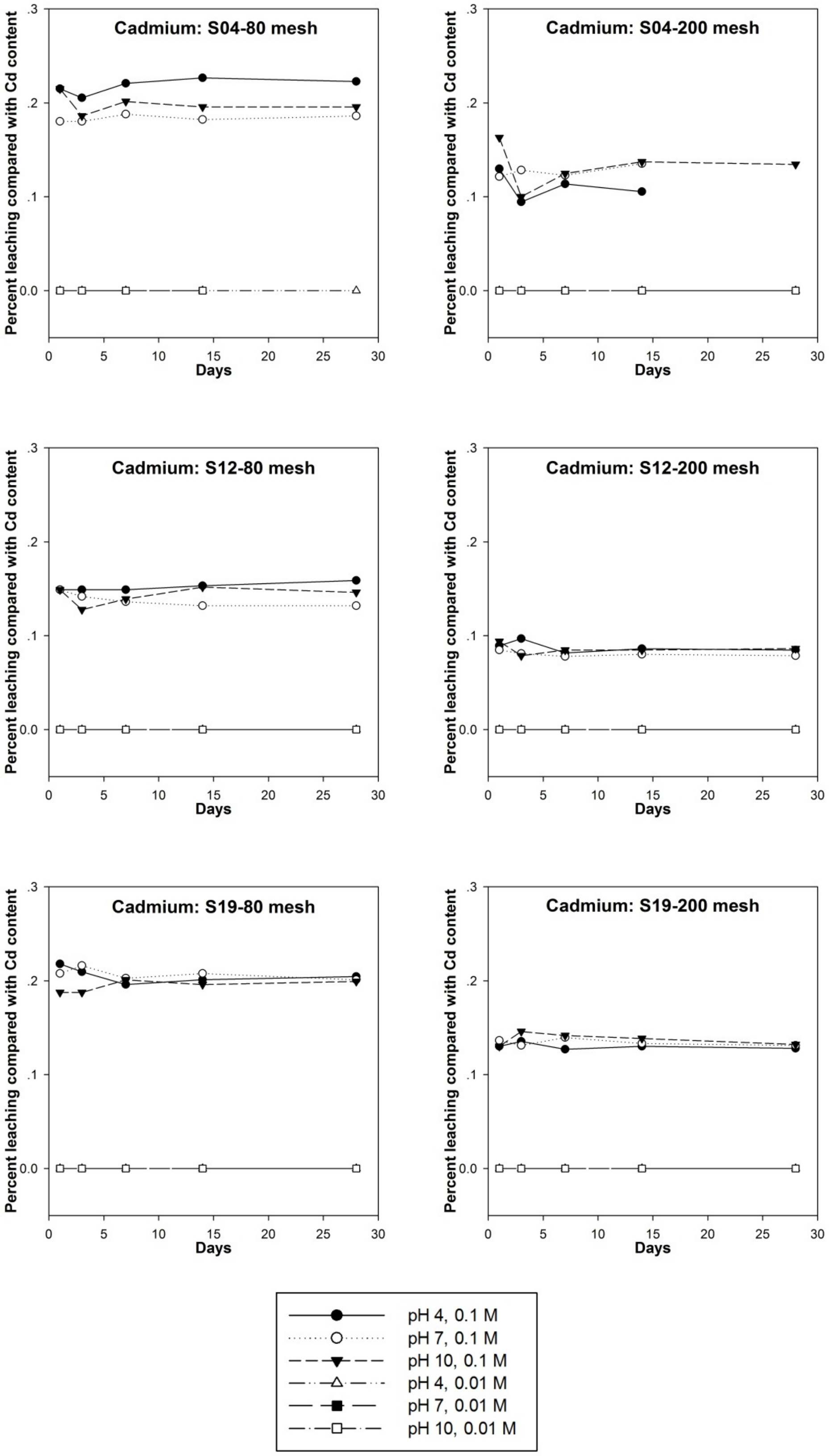

Figure S1: Percent leached concentrations of cadmium from soil samples number S04, S12, and S19 in 80-mesh and 200-mesh size in test time of 28 days.

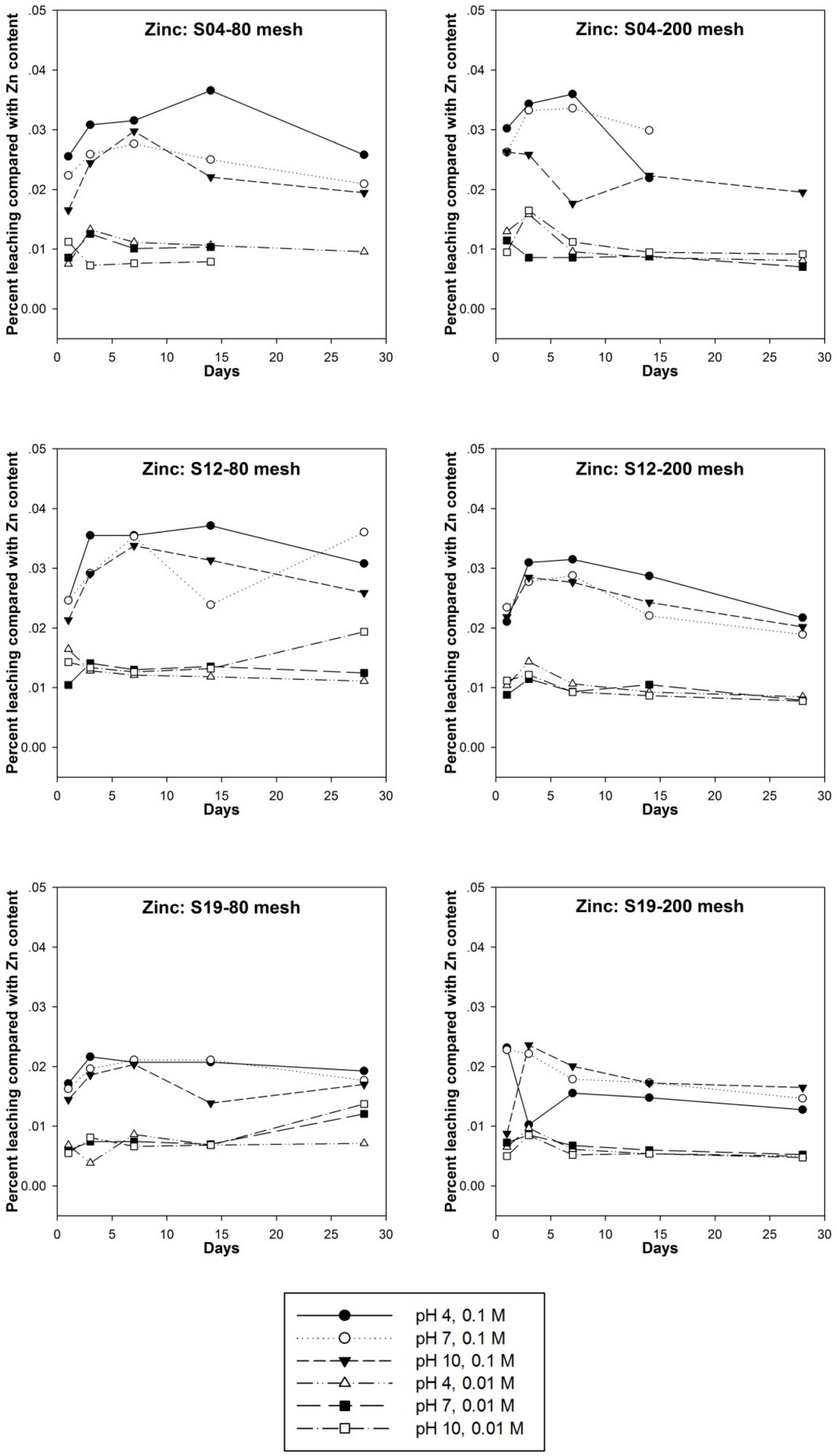

Figure S2: Percent leached concentrations of zinc from soil samples number S04, S12, and S19 in 80-mesh and 200-mesh size in test time of 28 days.

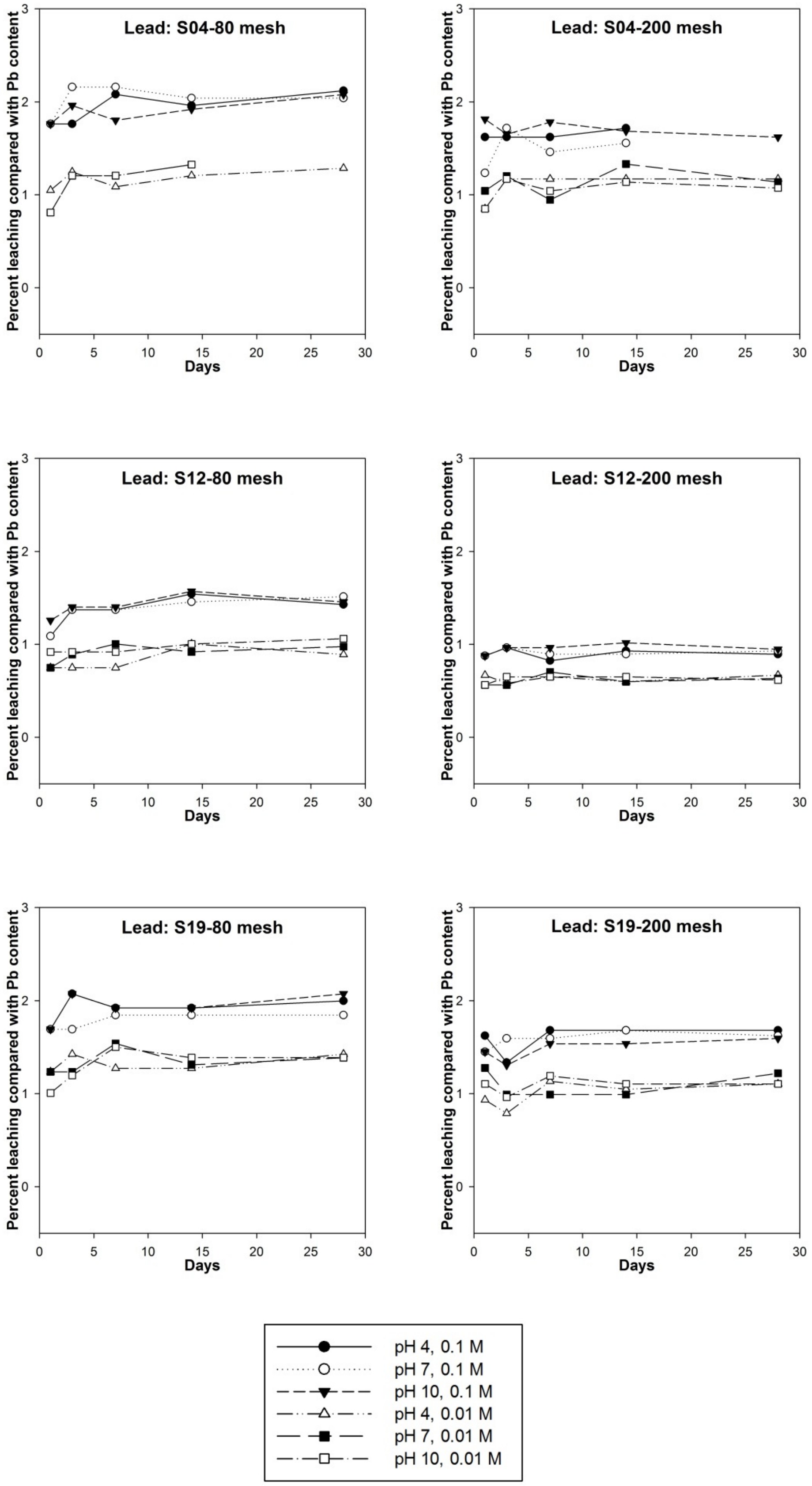

Figure S3: Percent leached concentrations of lead from soil samples number S04, S12, and S19 in 80-mesh and 200-mesh size in test time of 28 days.

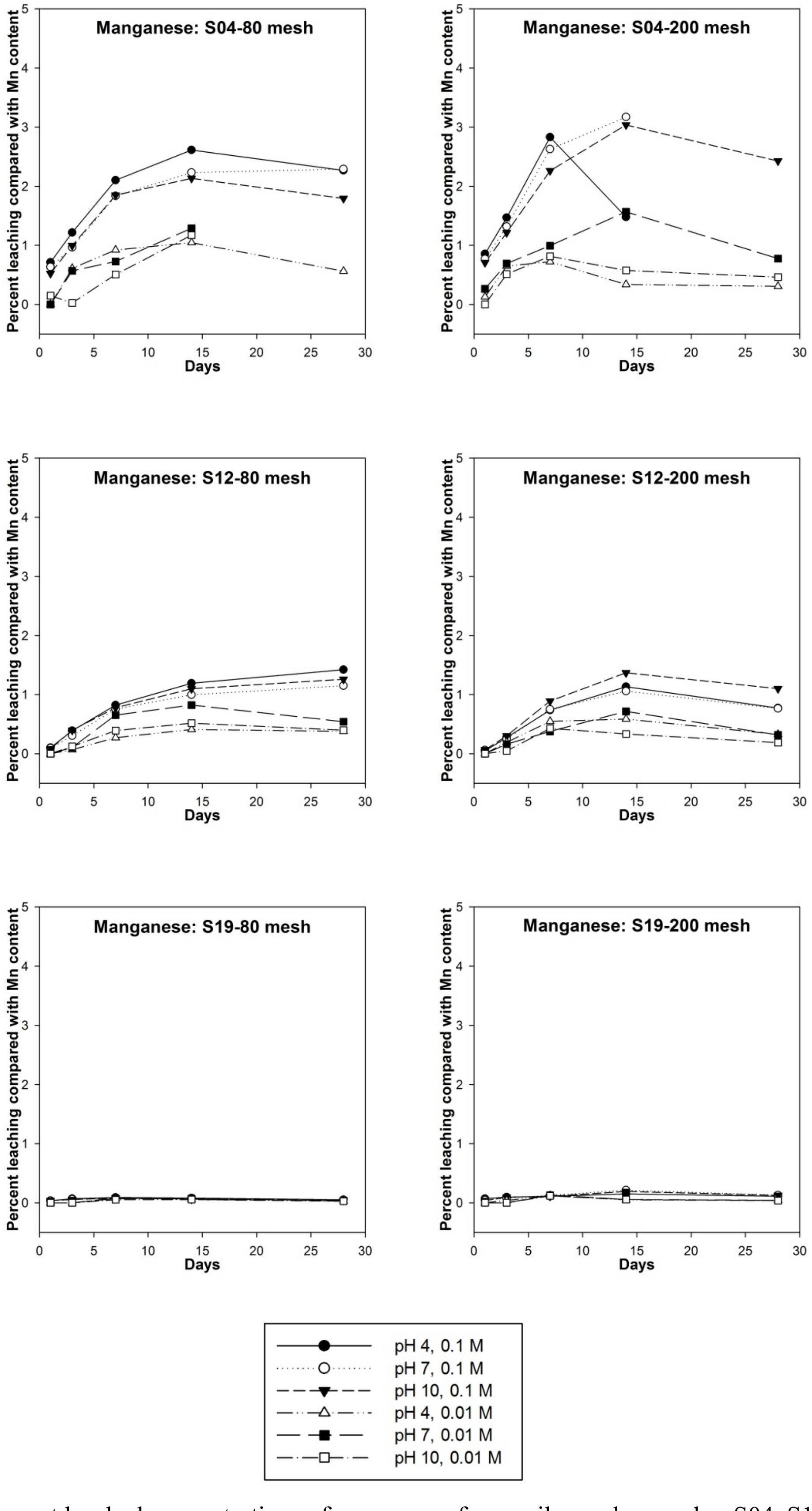

Figure S4: Percent leached concentrations of manganese from soil samples number S04, S12, and S19 in 80-mesh and 200-mesh size in test time of 28 day.
